# Crystal structure of the mixed-metal tris­ulfide BaCu_1/3_Ta_2/3_S_3_

**DOI:** 10.1107/S2056989017005266

**Published:** 2017-04-18

**Authors:** Kejun Bu, Jianqiao He, Dong Wang, Chong Zheng, Fuqiang Huang

**Affiliations:** a State Key Laboratory of High Performance Ceramics and Superfine Microstructure, Shanghai Institute of Ceramics, Chinese Academy of Sciences, Shanghai 200050, People’s Republic of China; b School of Materials Science and Engineering, Shanghai University, Shangda Road, No. 99, Shanghai 200444, People’s Republic of China; c Department of Chemistry and Biochemistry, Northern Illinois University, USA

**Keywords:** crystal structure, quasi-one-dimensional structure, mixed-metal tris­ulfide

## Abstract

The mixed-metal title compound, BaCu_1/3_Ta_2/3_S_3_ [barium copper(II) tantalum(V) tris­ulfide], was prepared through solid-state reactions. The crystal structure adopts the BaTaS_3_ structure type and consists of face-sharing [*M*S_6_] (*M* = Ta,Cu) octa­hedra (point-group symmetry -3*m*.) that are condensed into infinite chains along [001]. Adjacent chains are linked through the barium cations (site symmetry -6*m*2), which exhibit a coordination number of twelve. The *M* site is occupied by 2/3 of Ta^V^ and 1/3 of Cu^II^, whereby the average *M*—S distances are slightly longer than those of ordered BaTaS_3_. The classical charge balance of the title compound can be represented by [Ba^2+^] [(Ta/Cu)^4+^] [S^2−^]_3_.

## Abstract 2

In the structure of BaCu_1/3_Ta_2/3_S_3_, the Cu and Ta atoms are occupationally disordered on the same site in a ratio of 1/3:2/3.

## Chemical context   

Barium vanadium tris­ulfide, BaVS_3_ (Takano *et al.*, 1977 ▸), with which BaTaS_3_ (Gardner *et al.*, 1969 ▸) crystallizes isotypically in space group *P*6_3_/*mmc*, has a chain structure. The observed conductivity was attributed to the formation of conduction bands *via* vanadium⋯vanadium *d*-orbital overlap. It shows three phase transitions and exhibits a number of intriguing physical properties (Nakamura *et al.*, 1994 ▸). While both BaVS_3_ and BaTaS_3_ are composed of the same type of linear chains, BaTaS_3_ shows metallic conductivity and a Curie–Weiss behaviour of the magnetic susceptibility (Gardner *et al.*, 1969 ▸). To explore the physical properties of BaTaS_3_ and related compounds, we have introduced copper and studied mixed-metal phases Ba(Ta/Cu)S_3_. Here we report on the synthesis and structural characterization of the mixed-metal tris­ulfide with composition BaCu_1/3_Ta_2/3_S_3_.

## Structural commentary   

**Figure 1:**
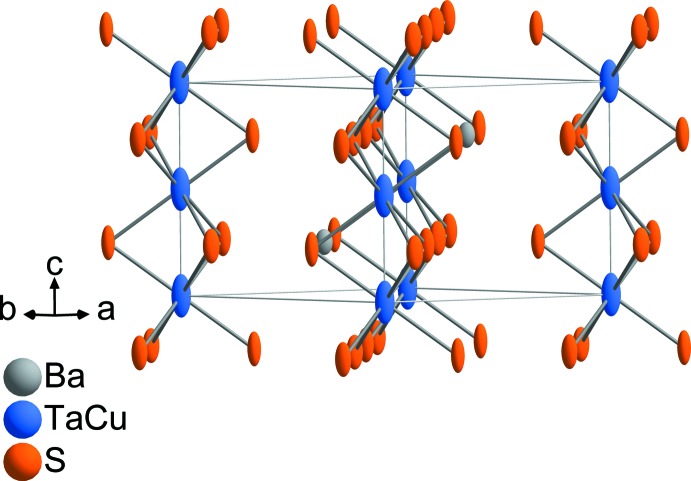
Face-sharing of *M*S_6_ (*M* = Cu, Ta) octa­hedra in the structure of BaCu_1/3_Ta_2/3_S_3_. Displacement ellipsoids are drawn at the 50% probability level.

**Figure 2:**
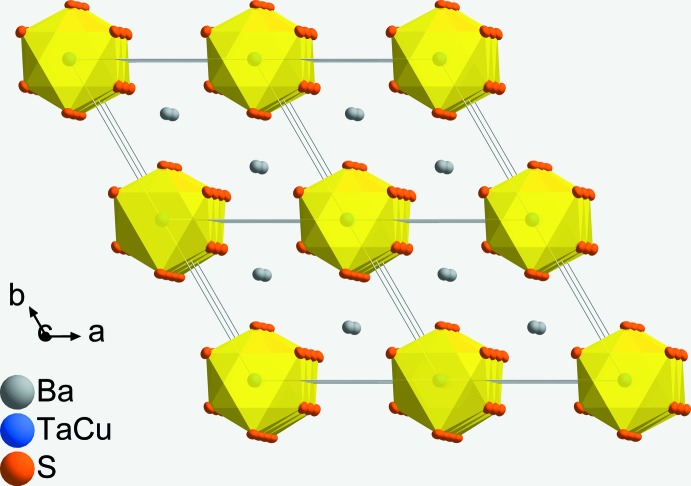
The crystal structure of BaCu_1/3_Ta_2/3_S_3_, viewed down [001].

BaCu_1/3_Ta_2/3_S_3_ adopts the BaTaS_3_ structure type in space group *P*6_3_/*mmc*. A detailed description of this structure type has been given previously (Gardner *et al.*, 1969 ▸). The asymmetric unit of BaCu_1/3_Ta_2/3_S_3_ contains one Ba site (Wyckoff position 2*c*), one mixed-occupied (Cu/Ta) site (2*a*) and one S site (6*h*). The structure contains face-sharing octa­hedral [*M*S_6_] (*M* = Cu, Ta) units, which construct infinite chains along [001] (Fig. 1 ▸). In the crystal structure, these chains are linked through Ba cations (coordination number 12) to adjacent chains (Fig. 2 ▸).

The *M* site is occupationally disordered and contains 1/3 Cu and 2/3 Ta. It is surrounded by six S atoms with an *M*—S bond length of 2.475 (4) Å, which is slightly longer than that of ordered BaTaS_3_ (2.461 Å; Gardner *et al.*, 1969 ▸). This trend is in agreement with the different ionic radii of Ta (0.64 Å for Ta^V^ with coordination number of six) and Cu^II^ (0.73 Å) using the data provided by Shannon (1976 ▸).

The (Cu,Ta)⋯(Cu,Ta) distance within a chain is 2.9159 (3) Å, which is much shorter than the inter­chain (Cu,Ta)⋯(Cu,Ta) distance of 6.8437 (18) Å. The Ba—S inter­actions between adjacent metal sulfide chains are reflected by one shorter [3.422 (6) Å] and one longer distance [3.523 (3) Å], in good agreement with those found in other barium tantalum sulfides (Onoda & Saeki, 1989 ▸).

The classical charge balance of the title compound can be represented by the formula [Ba^2+^] [(Ta/Cu)^4+^] [S^2−^]_3_.

## Synthesis and crystallization   

The title compound was prepared using solid-state reactions between the elements Cu, Ta, S and BaS. Ta powder (99.999%, Alfa Aesar Puratronic), Cu powder (99.999%, Alfa Aesar Puratronic), S powder (99.999%, Alfa Aesar Puratronic), and BaS powder (99.999%, Alfa Aesar Puratronic) were mixed in a fused-silica tube in an Ta:Cu:S:BaS molar ratio of 0.67:0.33:2:1. The tube was evacuated to 0.1 Pa, sealed and heated gradually (60 K h^−1^) to 973 K, where it was kept for 2 d. The tube was then cooled to 673 K at a rate of 3 K h^−1^ and then quenched to room temperature. The crystals are stable in air and alcohol.

**Figure 3:**
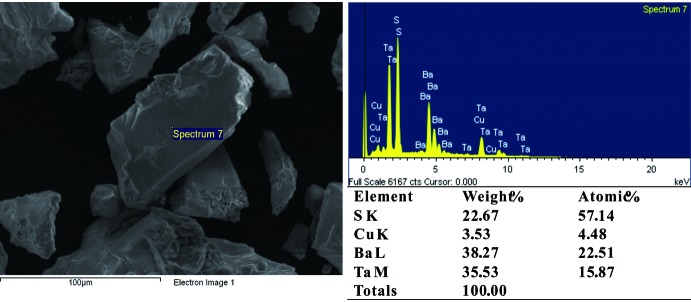
SEM image and EDX spectrum of BaCu_1/3_Ta_2/3_S_3_.

Scanning electron microscopy (SEM) images of selected crystals were taken on a Hitachi S-4800 microscope equipped with an electron microprobe analyzer for a semiqu­anti­tative elemental analysis in the energy dispersive X-ray spectroscopy (EDX) mode. The presence of both copper and tantalum was confirmed (Fig. 3 ▸).

## Refinement   

Crystal data, data collection and structure refinement details are summarized in Table 1 ▸. The refinement of the model with occupational disorder on the *M* site resulted in a significant decrease of the reliability factors in comparison with a fully occupied Ta site (*R*1 = 0.73, *wR* = 0.197). No evidence, *e.g*. in the form of superstructure reflections, was found for an ordering of this site and thus a statistically disordered model was considered. In the final model, atoms of the disordered site were restrained to have the same displacement parameters, with a fixed Cu:Ta ratio of 1/3:2/3 required for charge neutrality and in good agreement with the EDX measurement. The remaining maximum and minimum electron densities are located 1.06 Å from the (Cu,Ta)1 site and 1.96 Å from the S1, respectively.

## Supplementary Material

Crystal structure: contains datablock(s) global, I. DOI: 10.1107/S2056989017005266/wm5362sup1.cif

Structure factors: contains datablock(s) I. DOI: 10.1107/S2056989017005266/wm5362Isup3.hkl

CCDC reference: 1542709

Additional supporting information:  crystallographic information; 3D view; checkCIF report

## Figures and Tables

**Table 1:** Experimental details

Crystal data	
Chemical formula	BaCu_1/3_Ta_2/3_S_3_
*M*_r_	375.14
Crystal system, space group	Hexagonal, *P*6_3_/*m* *m* *c*
Temperature (K)	297
*a*, *c* (Å)	6.8350 (6), 5.8318 (5)
*V* (Å^3^)	235.94 (5)
*Z*	2
Radiation type	Mo *K*α
μ (mm^−1^)	26.34
Crystal size (mm)	0.04 × 0.03 × 0.01

Data collection	
Diffractometer	Bruker D8 QUEST
Absorption correction	Multi-scan (*SADABS*; Bruker, 2015 ▸)
*T*_min_, *T*_max_	0.44, 0.86
No. of measured, independent and observed [*I* > 2σ(*I*)] reflections	4312, 125, 106
*R*_int_	0.042
(sin θ/λ)_max_ (Å^−1^)	0.645

Refinement	
*R*[*F* ^2^ > 2σ(*F* ^2^)], *wR*(*F* ^2^), *S*	0.043, 0.113, 1.25
No. of reflections	125
No. of parameters	11
No. of restraints	2
Δρ_max_, Δρ_min_ (e Å^−3^)	1.50, −1.50

Computer programs: *APEX3* and *SAINT* (Bruker, 2015 ▸), *SHELXT* (Sheldrick, 2015*a* ▸), *SHELXL2014/7* (Sheldrick, 2015*b* ▸), *DIAMOND* (Brandenburg, 2007 ▸) and *publCIF* (Westrip, 2010 ▸).

## supplementary crystallographic information

### Crystal data

**Table:**

BaCu_0.33_Ta_0.67_S_3_	*D*_x_ = 5.280 Mg m^−^^3^
*M**_r_* = 375.14	Mo *K*α radiation, λ = 0.71073 Å
Hexagonal, *P*6_3_/*m**m**c*	Cell parameters from 1738 reflections
*a* = 6.8350 (6) Å	θ = 3.4–25.9°
*c* = 5.8318 (5) Å	µ = 26.34 mm^−^^1^
*V* = 235.94 (5) Å^3^	*T* = 297 K
*Z* = 2	Plate, black
*F*(000) = 325	0.04 × 0.03 × 0.01 mm

### Data collection

**Table:**

Bruker D8 QUEST diffractometer	106 reflections with *I* > 2σ(*I*)
Detector resolution: 10.4167 pixels mm^-1^	*R*_int_ = 0.042
phi and ω scans	θ_max_ = 27.3°, θ_min_ = 3.4°
Absorption correction: multi-scan (*SADABS*; Bruker, 2015)	*h* = −8→8
*T*_min_ = 0.44, *T*_max_ = 0.86	*k* = −8→8
4312 measured reflections	*l* = −7→6
125 independent reflections	

### Refinement

**Table:**

Refinement on *F*^2^	11 parameters
Least-squares matrix: full	2 restraints
*R*[*F*^2^ > 2σ(*F*^2^)] = 0.043	*w* = 1/[σ^2^(*F*_o_^2^) + (0.0503*P*)^2^ + 4.8137*P*] where *P* = (*F*_o_^2^ + 2*F*_c_^2^)/3
*wR*(*F*^2^) = 0.113	(Δ/σ)_max_ < 0.001
*S* = 1.25	Δρ_max_ = 1.50 e Å^−^^3^
125 reflections	Δρ_min_ = −1.50 e Å^−^^3^

### Special details

**Table:**

Geometry. All esds (except the esd in the dihedral angle between two l.s. planes) are estimated using the full covariance matrix. The cell esds are taken into account individually in the estimation of esds in distances, angles and torsion angles; correlations between esds in cell parameters are only used when they are defined by crystal symmetry. An approximate (isotropic) treatment of cell esds is used for estimating esds involving l.s. planes.

### Fractional atomic coordinates and isotropic or equivalent isotropic displacement parameters (Å^2^)

**Table:**

	*x*	*y*	*z*	*U*_iso_*/*U*_eq_	Occ. (<1)
Ba	0.6667	0.3333	0.75	0.0341 (7)	
Ta	0	0	0.5	0.0707 (12)	0.6666 (8)
Cu	0	0	0.5	0.0707 (12)	0.3334 (18)
S	0.1689 (4)	0.3378 (8)	0.75	0.0449 (15)	

### Atomic displacement parameters (Å^2^)

**Table:**

	*U*^11^	*U*^22^	*U*^33^	*U*^12^	*U*^13^	*U*^23^
Ba	0.0246 (7)	0.0246 (7)	0.0529 (13)	0.0123 (4)	0	0
Ta	0.0306 (8)	0.0306 (8)	0.151 (3)	0.0153 (4)	0	0
Cu	0.0306 (8)	0.0306 (8)	0.151 (3)	0.0153 (4)	0	0
S	0.0217 (18)	0.015 (2)	0.096 (4)	0.0074 (10)	0	0

### Geometric parameters (Å, º)

**Table:**

Ba—S^i^	3.4176 (3)	Ta—S	2.475 (4)
Ba—S^ii^	3.4176 (3)	Ta—S^xiii^	2.475 (4)
Ba—S^iii^	3.4176 (3)	Ta—S^vii^	2.475 (4)
Ba—S^iv^	3.4176 (3)	Ta—S^xiv^	2.475 (4)
Ba—S	3.4176 (3)	Ta—Ta^xv^	2.9159 (3)
Ba—S^v^	3.4176 (3)	Ta—Cu^xvi^	2.9159 (3)
Ba—S^vi^	3.506 (3)	Ta—Cu^xv^	2.9159 (3)
Ba—S^vii^	3.506 (3)	Ta—Ta^xvi^	2.9159 (3)
Ba—S^viii^	3.506 (3)	S—Cu^xv^	2.475 (4)
Ba—S^ix^	3.506 (3)	S—Ta^xv^	2.475 (4)
Ba—S^x^	3.506 (3)	S—Ba^xvii^	3.4176 (3)
Ba—S^xi^	3.506 (3)	S—Ba^vi^	3.506 (3)
Ta—S^xii^	2.475 (4)	S—Ba^ix^	3.506 (3)
Ta—S^iii^	2.475 (4)		
			
S^i^—Ba—S^ii^	60.89 (16)	S^xii^—Ta—S^iii^	180.0
S^i^—Ba—S^iii^	120.0010 (10)	S^xii^—Ta—S	91.18 (10)
S^ii^—Ba—S^iii^	59.11 (17)	S^iii^—Ta—S	88.82 (10)
S^i^—Ba—S^iv^	59.11 (17)	S^xii^—Ta—S^xiii^	88.82 (10)
S^ii^—Ba—S^iv^	120.0010 (10)	S^iii^—Ta—S^xiii^	91.18 (10)
S^iii^—Ba—S^iv^	179.11 (16)	S—Ta—S^xiii^	180.0
S^i^—Ba—S	179.11 (17)	S^xii^—Ta—S^vii^	88.82 (10)
S^ii^—Ba—S	120.0000 (10)	S^iii^—Ta—S^vii^	91.18 (10)
S^iii^—Ba—S	60.89 (17)	S—Ta—S^vii^	91.18 (10)
S^iv^—Ba—S	119.9990 (10)	S^xiii^—Ta—S^vii^	88.82 (10)
S^i^—Ba—S^v^	120.0000 (10)	S^xii^—Ta—S^xiv^	91.18 (10)
S^ii^—Ba—S^v^	179.11 (17)	S^iii^—Ta—S^xiv^	88.82 (10)
S^iii^—Ba—S^v^	119.9990 (10)	S—Ta—S^xiv^	88.82 (10)
S^iv^—Ba—S^v^	60.89 (17)	S^xiii^—Ta—S^xiv^	91.18 (10)
S—Ba—S^v^	59.11 (17)	S^vii^—Ta—S^xiv^	180.0
S^i^—Ba—S^vi^	89.75 (5)	S^xii^—Ta—Ta^xv^	126.10 (7)
S^ii^—Ba—S^vi^	118.88 (3)	S^iii^—Ta—Ta^xv^	53.90 (7)
S^iii^—Ba—S^vi^	118.88 (3)	S—Ta—Ta^xv^	53.90 (7)
S^iv^—Ba—S^vi^	61.40 (8)	S^xiii^—Ta—Ta^xv^	126.10 (7)
S—Ba—S^vi^	89.75 (5)	S^vii^—Ta—Ta^xv^	126.10 (7)
S^v^—Ba—S^vi^	61.40 (8)	S^xiv^—Ta—Ta^xv^	53.90 (7)
S^i^—Ba—S^vii^	118.88 (3)	S^xii^—Ta—Cu^xvi^	53.90 (7)
S^ii^—Ba—S^vii^	89.75 (5)	S^iii^—Ta—Cu^xvi^	126.10 (7)
S^iii^—Ba—S^vii^	61.40 (8)	S—Ta—Cu^xvi^	126.10 (7)
S^iv^—Ba—S^vii^	118.88 (3)	S^xiii^—Ta—Cu^xvi^	53.90 (7)
S—Ba—S^vii^	61.40 (8)	S^vii^—Ta—Cu^xvi^	53.90 (7)
S^v^—Ba—S^vii^	89.75 (5)	S^xiv^—Ta—Cu^xvi^	126.10 (7)
S^vi^—Ba—S^vii^	147.76 (6)	Ta^xv^—Ta—Cu^xvi^	180.0
S^i^—Ba—S^viii^	118.88 (3)	S^xii^—Ta—Cu^xv^	126.10 (7)
S^ii^—Ba—S^viii^	89.75 (5)	S^iii^—Ta—Cu^xv^	53.90 (7)
S^iii^—Ba—S^viii^	61.40 (8)	S—Ta—Cu^xv^	53.90 (7)
S^iv^—Ba—S^viii^	118.88 (3)	S^xiii^—Ta—Cu^xv^	126.10 (7)
S—Ba—S^viii^	61.40 (8)	S^vii^—Ta—Cu^xv^	126.10 (7)
S^v^—Ba—S^viii^	89.75 (5)	S^xiv^—Ta—Cu^xv^	53.90 (7)
S^vi^—Ba—S^viii^	57.48 (11)	Ta^xv^—Ta—Cu^xv^	0
S^vii^—Ba—S^viii^	112.55 (14)	Cu^xvi^—Ta—Cu^xv^	180.0
S^i^—Ba—S^ix^	89.75 (5)	S^xii^—Ta—Ta^xvi^	53.90 (7)
S^ii^—Ba—S^ix^	118.88 (3)	S^iii^—Ta—Ta^xvi^	126.10 (7)
S^iii^—Ba—S^ix^	118.88 (3)	S—Ta—Ta^xvi^	126.10 (7)
S^iv^—Ba—S^ix^	61.40 (8)	S^xiii^—Ta—Ta^xvi^	53.90 (7)
S—Ba—S^ix^	89.75 (5)	S^vii^—Ta—Ta^xvi^	53.90 (7)
S^v^—Ba—S^ix^	61.40 (8)	S^xiv^—Ta—Ta^xvi^	126.10 (7)
S^vi^—Ba—S^ix^	112.55 (14)	Ta^xv^—Ta—Ta^xvi^	180.0
S^vii^—Ba—S^ix^	57.48 (11)	Cu^xvi^—Ta—Ta^xvi^	0
S^viii^—Ba—S^ix^	147.76 (6)	Cu^xv^—Ta—Ta^xvi^	180.0
S^i^—Ba—S^x^	61.40 (8)	Ta—S—Cu^xv^	72.2
S^ii^—Ba—S^x^	61.40 (8)	Ta—S—Ta^xv^	72.19 (13)
S^iii^—Ba—S^x^	89.75 (5)	Cu^xv^—S—Ta^xv^	0
S^iv^—Ba—S^x^	89.75 (5)	Ta—S—Ba	89.64 (7)
S—Ba—S^x^	118.88 (3)	Cu^xv^—S—Ba	89.64 (7)
S^v^—Ba—S^x^	118.88 (3)	Ta^xv^—S—Ba	89.64 (7)
S^vi^—Ba—S^x^	57.48 (11)	Ta—S—Ba^xvii^	89.64 (7)
S^vii^—Ba—S^x^	147.76 (6)	Cu^xv^—S—Ba^xvii^	89.64 (7)
S^viii^—Ba—S^x^	57.48 (11)	Ta^xv^—S—Ba^xvii^	89.64 (7)
S^ix^—Ba—S^x^	147.76 (6)	Ba—S—Ba^xvii^	179.11 (16)
S^i^—Ba—S^xi^	61.40 (8)	Ta—S—Ba^vi^	159.82 (13)
S^ii^—Ba—S^xi^	61.40 (8)	Cu^xv^—S—Ba^vi^	87.629 (7)
S^iii^—Ba—S^xi^	89.75 (5)	Ta^xv^—S—Ba^vi^	87.629 (7)
S^iv^—Ba—S^xi^	89.75 (5)	Ba—S—Ba^vi^	90.25 (5)
S—Ba—S^xi^	118.88 (3)	Ba^xvii^—S—Ba^vi^	90.25 (5)
S^v^—Ba—S^xi^	118.88 (3)	Ta—S—Ba^ix^	87.629 (7)
S^vi^—Ba—S^xi^	147.76 (6)	Cu^xv^—S—Ba^ix^	159.82 (13)
S^vii^—Ba—S^xi^	57.48 (11)	Ta^xv^—S—Ba^ix^	159.82 (13)
S^viii^—Ba—S^xi^	147.76 (6)	Ba—S—Ba^ix^	90.25 (5)
S^ix^—Ba—S^xi^	57.48 (11)	Ba^xvii^—S—Ba^ix^	90.25 (5)
S^x^—Ba—S^xi^	112.55 (13)	Ba^vi^—S—Ba^ix^	112.55 (13)

Symmetry codes: (i) *x*+1, *y*, *z*; (ii) −*y*+1, *x*−*y*, *z*; (iii) −*x*+*y*, −*x*, *z*; (iv) −*x*+*y*+1, −*x*+1, *z*; (v) −*y*+1, *x*−*y*+1, *z*; (vi) −*x*+1, −*y*+1, −*z*+2; (vii) *y*, −*x*+*y*, −*z*+1; (viii) *y*, −*x*+*y*, −*z*+2; (ix) −*x*+1, −*y*+1, −*z*+1; (x) *x*−*y*+1, *x*, −*z*+2; (xi) *x*−*y*+1, *x*, −*z*+1; (xii) *x*−*y*, *x*, −*z*+1; (xiii) −*x*, −*y*, −*z*+1; (xiv) −*y*, *x*−*y*, *z*; (xv) −*x*, −*y*, *z*+1/2; (xvi) −*x*, −*y*, *z*−1/2; (xvii) *x*−1, *y*, *z*.

## References

[bb1] Brandenburg, K. (2007). *DIAMOND*. Crystal Impact GbR, Bonn, Germany.

[bb2] Bruker (2015). *APEX3*, *SAINT* and *SADABS*. Bruker AXS Inc., Madison, Wisconsin, USA.

[bb3] Gardner, R. A., Vlasse, M. & Wold, A. (1969). *Inorg. Chem.* **8**, 2784–2787.

[bb4] Nakamura, M., Sekiyama, A., Namatame, H., Fujimori, A., Yoshihara, H., Ohtani, T., Misu, A. & Takano, M. (1994). *Phys. Rev. B*, **49**, 16191–16201. 10.1103/physrevb.49.16191 10010765

[bb5] Onoda, M. & Saeki, M. (1989). *Mater. Res. Bull.* **24**, 625–631.

[bb6] Shannon, R. D. (1976). *Acta Cryst.* A**32**, 751–767.

[bb7] Sheldrick, G. M. (2015*a*). *Acta Cryst.* A**71**, 3–8.

[bb8] Sheldrick, G. M. (2015*b*). *Acta Cryst.* C**71**, 3–8.

[bb9] Takano, M., Kosugi, H., Nakanishi, N., Shimada, M., Wada, T. & Koizumi, M. (1977). *J. Phys. Soc. Jpn*, **43**, 1101–1102.

[bb10] Westrip, S. P. (2010). *J. Appl. Cryst.* **43**, 920–925.

